# Growth Factor Receptors and Apoptosis Regulators: Signaling Pathways, Prognosis, Chemosensitivity and Treatment Outcomes of Breast Cancer

**DOI:** 10.4137/bcbcr.s2492

**Published:** 2009-08-17

**Authors:** Siddik Sarkar, Mahitosh Mandal

**Affiliations:** 1 School of Medical Science and Technology, Indian Institute of Technology Kharagpur, Kharagpur-721302, India

**Keywords:** prognostic markers, estrogen, growth factor receptor, apoptosis, chemotherapy, mitogen-activated protein kinase

## Abstract

Biomarkers of breast cancer are necessary for prognosis and prediction to chemotherapy. Prognostic biomarkers provide information regarding outcome irrespective of therapy, while predictive biomarkers provide information regarding response to therapy. Candidate prognostic biomarkers for breast cancers are growth factor receptors, steroid receptors, Ki-67, cyclins, urokinase plasminogen activator, p53, p21, pro- and anti-apoptotic factors, BRCA1 and BRCA2. But currently, the predictive markers are Estrogen and Progesterone receptors responding to endocrine therapy, and HER-2 responding to herceptin. But there are numerous breast cancer cases, where tamoxifen is ineffective even after estrogen receptor positivity. This lead to search of new prognostic and predictive markers and the number of potential markers is constantly increasing due to proteomics and genomics studies. However, most biomarkers individually have poor sensitivity or specificity, or other clinical value. It can be resolved by studying various biomarkers simultaneously, which will help in better prognosis and increasing sensitivity for chemotherapeutic agents. This review is focusing on growth factor receptors, apoptosis markers, signaling cascades, and their correlation with other associated biomarkers in breast cancers. As our knowledge regarding molecular biomarkers for breast cancer increases, prognostic indices will be developed that combine the predictive power of individual molecular biomarkers with specific clinical and pathologic factors. Rigorous comparison of these existing as well as emerging markers with current treatment selection is likely to see an escalation in an era of personalized medicines to ensure the breast cancer patients receive optimal treatment. This will also solve the treatment modalities and complications related to chemotherapeutic regimens.

## Introduction

Breast cancer is one of the most frequently diagnosed cancers affecting females. It is estimated that this disease will afflict one in eight females in America during their lifetime. It is estimated that occurrence of breast cancer is 26% of cancers from all sites. An estimated 40,910 (40,460 women, 450 men) breast cancer death rate are expected by 2007, accounting 15% death with respect to other sites.^1^ Breast cancer is characterized by higher expression of epidermal growth factor receptor (EGFR) and estrogen/progesterone receptor (ER/PR). It is also found that higher expression of vascular endothelial growth factor (VEGFR-2) is associated with metastasis and poor survival outcomes in breast cancer patients. The overexpression of either growth factors or its receptors leads to abnormal signaling pathways that contributes to the progression, invasion, and malignant phenotype. Patients suffering from breast carcinoma have a poor prognosis because of the lack of effective treatment strategies. Detection and identification of prognostic markers for predicting therapeutic response is very essential for breast cancer treatment. The knowledge of individual prognostic marker remains poorly understood at molecular level in breast cancer prognosis and prediction of particular treatment regimens and its overall survival. Most recently gene profiling helps in clustering and segregating the genes on the basis of prognosis of disease outcome and the correlation of various prognostic factors with metastasis, ER, HER-2 status.^2^,^3^ In a study of 295 patients diagnosed with stage I or II breast cancer, those classified as having a good prognosis profile had a 94.5 ± 2.6 overall 10-year survival rate compared to 54.6% ± 4.4% for those with a poor profile and the probability of remaining free of distant metastases was 50.6% ± 4.5% and 85.2% ± 4.3% in the group with poor and a good-prognosis signature^4^ and thus the gene profiling can be a more valuable prognostic factor in predicting the disease outcome than the standard clinical and histogical system of classifications based on axillary lymph node status, tumor size and tumor grade.^5^,^6^ Prognostic and currently the predictive markers respond to endocrine therapy (tamoxifen and raloxifene) are ER/PR and HER-2 for identifying breast cancer metastasis responding to herceptin.^7^ Unfortunately, a substantial number of tumors are intrinsically tamoxifen-resistant, despite estrogen-receptor positivity, and eventually almost all breast carcinomas acquire resistance towards tamoxifen. This may be due to activation or cross talk signaling of other growth factor/cell signaling pathways compensating the inhibition of upstream regulators of particular pathways. It can be overcome by designing drugs inhibiting two pathways or bidirectional pathways. Tumor resistance is also acquired due to the overexpression of ATP-binding cassette (ABC) transporters.^8^ This can be overcome by designing chemotherapeutic drugs inhibiting ABC transporters, and thus overcoming multidrug resistance (MDR) and sensitizing the tumors for chemotherapeutic drugs. Thus, the detailed understanding of cross talk signaling pathways between growth factor, steroid receptors and apoptosis regulators as well as MDR properties will lead to the development of new drugs that are sensitive and having fewer side effects.

### Receptors of breast cancer

Beast cancer patients with the same diagnostic and clinical prognostic profile can have markedly different clinical and survival out-comes. This difference is possibly caused by the limitation of our classification on the basis of morphology without molecular classification. The differential gene expression patterns generated by microarray technology broadly classified breast cancer into two sub types ER positive (ER+/ErbB-2−) and ER negative (ER−/ErbB-2+). ER− tumors are further grouped into basal-like and ErbB-2 subtypes, both with poor prognosis. In contrast, ER+ breast cancers could be classified into luminal A and luminal B subtypes with significantly distinct prognosis, luminal A tumors displayed favorable outcome, whereas survival of patients with luminal B tumors was poor and comparable to those of the ER negative ErbB-2 and basal subtypes.^2^ The molecular classifications are found to be strongly associated with ER status and moderately associated with grade, but not associated with menopausal status, nodal status, or tumor size.^3^

### Estrogen receptor

**Figure 1.**
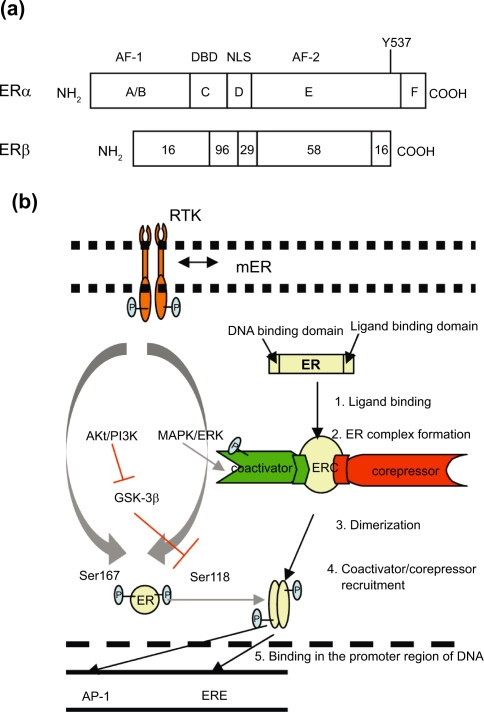
Homology of ERα and ERβ **a**) Estrogen receptor and cross-talk signaling with RTK in breast cancer. **b**) Estrogen leads to transcription of cell proliferation gene via classical pathway (black arrow). Cross talk and bidirectional (gray arrows) signaling between mER, RTK, mER and downstream phosphorylation of Ser118 and 167 by MAPK/ERK and PI3-K/Akt pathway respectively leads to activation of estrogen regulated genes. GSK-3β inhibits (blocking red arrows) phosphorylation of Ser118 which is further inactivated by PI3-K/AKT. **Abbreviations:** DBD, DNA binding domain; NLS, Nuclear localization sequence.

ER can be classified into two forms ERα and ERβ, and mainly ERα are over-expressed around 70% breast cancer cases.^9^,^10^ It was found that there is alter or increase in expression of α and β forms of ER in tumor as compared to normal tissues^11^ and may contribute to amplified estrogen response. Both α and β forms are homologous especially in DNA binding domain (96%) and hormone dependent activation function domain (AF-2) (56%). They have the least homology (16%) in hormone independent activation function domain (AF-1) (Fig. 1a), and probably the signaling cascade regulated by AF-1 may be responsible for endocrine irresponsive behavior of breast cancers expressing ERβ. Both ERα and ERβ activated estrogen response element (ERE) in response to estrogen and are inhibited with anti-estrogen.^12^ Estrogen binds to ER that lead to series of sequential events (Fig. 1b), finally binding to ERE present in the promoters of various target genes^13^ resulting in cell proliferation. ER complex can also bind to transciption factors, such as activator protein (AP-1) or Sp-1 that leads to chromatin modification by histone unwinding and helping in transcription of genes through alternative pathway. Estrogen regulated transcription at AP-1 site acts in two different directions in case of α and β forms of ER. Estrogen upregulates genes regulated by Ap-1 mediated transcription, whereas anti-estrogen downregulates it and reverse is observed in case of β form. Subsequently, two ERs signal in different ways depending upon ligands and response elements. The different function of selective estrogen receptor modulators (SERMs) in breast and uterine tissues may also be partially contributed by hormone independent AF-1 and hormone dependent AF-2. There are certain promoters that require both AF-1 and AF-2 activity, whereas in other promoters both AF-1 and AF-2 function independently. Promoters requiring AF-2 or both AF-1 and AF-2 upregulate genes in response to estrogen, whereas promoters requiring only AF-1 activity upregulate genes in response to anti-estrogen. It was observed by Tzukerman et al^14^ that tamoxifen (SERMs) does not activate AF-2, therefore, in tissues which require only AF-1 activation, tamoxifen is an agonist, whereas in tissues which require AF-2, or both AF-1 and AF-2, tamoxifen is an antagonist. Moreover, AF-1 region is involved in the interactions of ER with coactivators and repressors, further regulating the transcribed genes depending on the net ratio of coactivators and corepressors in particular tissue type.^15^,^16^ In addition a part of membrane ER (mER) is found associated with cell surface via caveolins.^17^ Although classical concepts had assigned priority to the nuclear actions of estrogen receptor, recent studies document the additional importance of mER in mediating the rapid effects of 17-β-estradiol (E2). It has no transmembrane and kinase domains and is known to initiate E2 rapid signals by forming a protein complex with many signaling molecules. It mediates its action via G-proteins, growth factor receptor-bound protein-2 (Grb2) and Grb2-associated binder 2 (Gab-2), receptor tyrosine kinase (RTK) like EGFR, insulin like growth factor (IGF) receptor (IGFR) and non receptor tyrosine kinase Src.^16^,^18^ Phosphorylation of Ser118 at nuclear ER by mitogen-activated protein kinase (MAPK1/3)/extracellular signal-regulated kinase (ERK)^17^,^19^ or phosphatidylinositol 3-kinase (PI3-K)/Akt pathways lead to upregulation of estrogen regulated genes, whereas glycogen synthase kinase 3 (GSK-3) inhibits the Ser118 phosphorylation of ER. The signaling and cross talk pathways between growth factors and estrogen signaling is bidirectional. It is observed that prolong exposure of anti-estrogen irreversibly causes cells to overexpress EGFR mainly HER-2, and also increased binding of EGFR with mER. This might lead to increased signaling pathway of particular growth factor, decreasing estrogen response and hence development of antiestrogen resistance.^20^ A full understanding of the mechanisms underlying these relationships, with the ultimate aim of abrogating specific steps, should lead to more-targeted strategies for treatment of hormone dependent-breast cancer.

### Epidermal growth factor receptor

**Figure 2.**
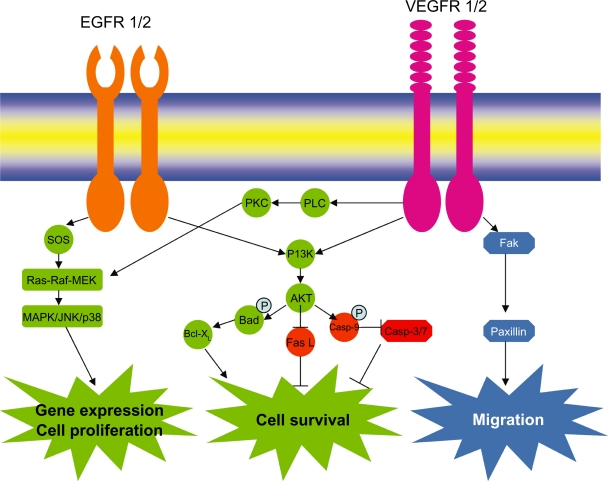
Epidermal growth factor receptor and Vascular endothelial growth factor receptor signaling in breast cancer.

Epidermal growth factor receptors (EGFRs) especially EGFR-1 and EGFR-2 (HER-2) play role in breast cancer progression. EGFR family of receptors includes four closely related receptor tyrosine kinases (RTKs): EGFR/HER-1 (ErbB-1), HER-2/neu (ErbB-2), HER-3 (ErbB-3) and HER-4 (ErbB-4). The highest degree of sequence homology (approximately 80% amino acid identity) between HER-2, EGFR and HER-4 lies in the tyrosine kinase domain, suggesting that this region is essential for the signaling function of these molecules. HER-3, by contrast, has four amino acids substitutions in its tyrosine kinase domain, leading to the suggestion that this receptor may have reduced or absent enzymatic activity and its activity is regained only after dimerization with other EGFRs.^21^ The crystal structure of HER receptors (HER-1, 3 and 4) are quite similar, having domain II–IV contacts. This prevents the intermolecular dimerization domain II from participating in homo- and heterodimerization. Ligand binding induces change in structural conformation; freeing domain II projection for receptor dimerization, that inturn activates RTK activity. There is no such contact between domain II–IV, making HER-2 always in active state and the preferred partners for other HER receptors.^22^–^24^ HER-2 overexpression has been reported to amplify EGFR signaling. HER-2/neu gene amplification is most common representing 25%–30%^25^ of breast cancer of ER− type and herceptin shows a promising clinical result with HER-2 positive breast cancers. Increase in HER-2 correlates with poor prognosis and increased propensity for metastasis. HER-2 amplification results in a 50 to 100-fold increase in the number of surface receptors on cancer cells compared to the normal mammary epithelium. HER-2 gene amplification and oncogenic mutations constitutively activate the HER-2 homodimeric tyrosine kinase.^26^ Elevated HER-2 activity can reduce the general growth factor dependence of HER-2 amplified cells though prolonged stimulation of the Ras/Raf/Mitogen-activated protein kinase (MAPK) pathway, and confers resistance to hormonal therapy.^27^ Upon dimerization of EGFRs by EGF and Hergulin (HRG) and receptor phosphorylation, Shc binds the receptor which further transmits the signal to GRB2, SOS and Ras which inturn leads to an increase in Raf kinase activity. Once activated, Raf phosphorylates and activates its major substrates mitogen activated extracellular signal regulated kinase activating kinases (MEK) family which includes MEK1 and MEK2, which in turn phosphorylate and activate the MAPKs (ERK1 and ERK2). MAPK family of kinases also includes p38^MAPK^ and c-Jun-N-terminal kinase (JNK). Inaddition to activation of Raf/MEK/ERK pathways, Ras and phosphorylated EGFR family members stimulates PI3-K activity by binding to p110 subunit and p85 subunit of PI3-K respectively (Fig. 2). Activated PI3-K phosphorylate substrates like phosphatidylinositol-4 phosphate and phosphatidylinosital 4, 5-bisphosphate to yield 3, 4-bisphosphate [PI(3,4)P_2_] and phosphatidylinositol 3,4,5-trisphosphate [PI(3,4,5)P_3_]. This in-turn recruits phosphoinositide dependent kinase-1 (PDK-1) and Akt family kinases to the plasma membrane and activate them. This signal transduction cascade initiated by PI3-K is referred to as the PI3-K/PDK1/Akt pathway. This cascade can prevent apoptosis.^28^,^29^ Although HER-2 gene amplification and protein overexpression have been extensively studied in breast cancer, data on EGFR overexpression in breast cancer are limited. EGFR overexpression and its activating-mutations at exons 18–21 in lung cancers have been extensively studied. It has been found that there is a strong response of tyrosine kinase inhibitors (e.g. erlotinib and gefitinib) to lung cancers exhibiting EGFR activating-mutations. These mutations selectively activate AKT and signal transduction and activator of transcription (STAT) signaling pathways, which promote cell survival. Gefitinib was found to bind the ATP pocket of catalytic domain of EGFR kinase, and thus inhibiting the anti-apoptotic signals transduced by mutant EGFR.^30^ Though there is lack of data of EGFR activating mutations associated with breast cancer, but EGFR overexpression has been reported to occur around 20%–36% breast cancer cases. These may be candidates for anti-EGFR therapies. Besides, there is strong synergy of EGFR with HER-2, so blocking both receptors may be critical. Interestingly, EGFR and HER-2 coexpression in breast cancer was recently associated with reduced overall survival (OS) and disease-free survival (DFS).^31^ It is also increasingly clear that the high cell-surface HER-2 density that accompanies gene amplification alters the normal equilibrium of EGFR dimers in favor of HER-2 containing heterodimers, thus altering ligand dependant signaling mechanisms. The oncogenic potency of heterodimers, EGFR/HER-2 for example, is significantly enhanced compared to EGFR homodimers by several processes that prolong receptor signaling activity. HER-2/neu + breast cancer is also characterized by higher expression of *MDR1*, *S100* calcium-binding protein P, fatty acid synthase, Ras related GTPases, *RAL-B*, *RAB6A*, fibronectin 1, and syndecan1 and lower expression of c-*kit* and c-*myc*.^32^ Moreover it is found that s-HER-2 elevation in early breast cancer correlates with the principal criteria of tumor aggressiveness and vascular invasion.^33^ It can act as independent prognostic factor enabling to identify patients likely to benefit from more aggressive treatments at the early stages of tumor metastasis.

### Vascular endothelial growth factor receptor

Breast cancer particularly metastatic breast cancer and angiogenesis is characterized by upregulation of VEGFR. It is a member of receptor tyrosine kinase family. VEGFR family of receptors consists of VEGFR-1 (flt-1), VEGFR-2 (KDR/flk-1) and VEGFR-3 (flt-4) and soluble form of VEGFR-1 (sVEGFR-1), an intensive negative counterpart of VEGF.^34^ VEGFR-1 and VEGFR-2 is mainly responsible for angiogenesis whereas VEGFR-3 is responsible for lymphogenesis. There are common as well as unique ligands for VEGFRs. VEGF-A, VEGF-B binds to VEGFR-1. VEGF-A, VEGF-C and VEGFD binds to VEGFR-2. VEGF-C, VEGF-D binds to VEGFR-3. VEGFR-1 and VEGFR-2 are structurally similar, consisting of an extracellular ligand binding domain with seven immunoglobulin (Ig)-like motifs, a single transmembrane domain and a juxtamembrane domain, a kinase domain split by a kinase insert, and a carboxyl terminus. Overall, there is 43.2% sequence homology between VEGFR-1 and VEGFR-2. The extracellular domain of VEGFR-1 and VEGFR-2 displays 33.3% homology and the cytoplasmic region displays 54.6% homology. The kinase domain of VEGFR-1 and VEGFR-2 represent the most conserved region with 70.1% homology. In contrast, the carboxyl terminus represents the most divergent region with only 28.1% sequence homology. This carboxyl terminus is responsible for cell signaling for angiogenesis. The difference in sequence of carboxyl terminus between VEGFR-1 from that of VEGFR-2 is responsible for the former kinase-impaired activity. Although ligands have strong affinity towards VEGFR-1 than VEGFR-2, but it is VEGFR-2 that responds to almost all of the cellular responses to VEGF. Upon VEGF binding, receptor dimerization takes place leading to transphosphorylation, an increase in the intrinsic catalytic activity and creation of binding sites on the RTKs to recruit cytoplasmic signaling proteins that activates ERK1/2 pathway and also PI3-K/Akt (Fig. 2) pathways. In the unstimulated form, the activation loop orients tyrosine residues toward the activation sites of the enzyme and thereby sterically prevents binding to ATP-Mg2+. After binding of ligand and dimerization of receptors, phosphorylation of tyrosine residues in the catalytic loop takes place that orients the inhibitory loop away from the active site enabling the RTK to bind ATP-Mg2+. It finally acts as the binding sites and phosphorylates SH2- and PTB- containing molecules. It is found that solid tumors are characterized by the presence of hypoxic pockets, and VEGFs/VEGFRs signaling are responsible for survival and aggressive nature of tumor cells. Hypoxia induced VEGF signaling and constitutively activates PI3-K, stimulates Akt activity and hence inhibits apoptosis and favors cell survival. There are also reports that VEGF signaling can induce the expression of anti-apoptotic Bcl-2, Bcl-xL and promotes cell survival.^35^,^36^ In addition, VEGFs/VEGFRs signaling promote cell adhesion and migration through activated αvβ3 integrin in breast cancer. Breast cancer cells mainly express VEGF-A, VEGF-B, VEGF-C and their receptors VEGFR-1, VEGFR-2 and neuropilin (NP-1/NP-2).^37^ VEGFR-1 is associated with poor prognosis, particularly those with node negative tumors, with high risk of metastasis and relapse.^38^,^39^ VEGFR-1 immunodetection may further be considered as a potential tool for evaluating tumor aggressiveness and therapeutic strategies in breast cancer whereas VEGFR-2 positive tumor surface was not correlated with survival or with metastasis risk and relapse.VEGFR-2 mainly functions in VEGF signaling whereas VEGFR-1 functions as a decoy receptor in VEGF signal regulation. VEGFR-3 is predominantly expressed in endothelium of lymphatic vessels. It promotes tumor lymphogenesis and lymphatic metastasis. VEGF-C/VEGFR-3 is also found in tumor tissues of breast cancer, and played a role in upregulating neural cell adhesion moleculer CNTN-1 through Src/p38 MAPK pathway.^40^ It promotes cell invasion via rearrangement of actin cytoskeleton. Neuropilins, which are receptors for the collapsin/semaphorin family responsible for neural cell guidance, binds to VEGF (VEGF165, but not to VEGF121). VEGF-A secretion by breast cancer cell lines MDA-MB-468, T-47D, MCF-7, HBL-100, and MW1 were about 2.3 to 37.3 ng/ml for 10^6^ cells for 48 hours, which was higher above the biological activity of VEGF-A (1–50 pg/ml),^37^ indicating the role of VEGF in breast cancer progression. VEGFR-2 phosphorylation was detected above the baseline in case of epithelial breast cancer cells than in endothelial breast cancer cell line, PAEC/KDR, without external VEGF stimulation. But there was a marked increase in VEGFR-2 phosphorylation following stimulation with VEGF. Weigand et al observed that VEGF-A-induced increase in Akt phosphorylation in T47D cells, while stimulation of MDA-MB-468, HBL-100, MCF-7 and MW1 with VEGF-A resulted in an increase in ERK1/2 phosphorylation. Therefore VEGF stimulation leads to phosphorylation of VEGFR-2 which in turn activates either ERK1/2 or PI3-K/Akt pathways.

### Apoptosis

**Figure 3.**
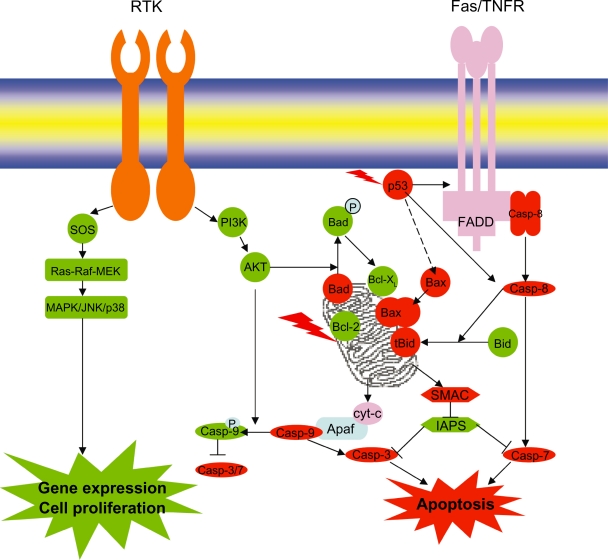
Apoptosis and croos-talk signaling with RTK in breast cancer.

Normal breast development is controlled by a balance between cell proliferation and apoptosis, and there is strong evidence that tumor growth is not just a result of uncontrolled proliferation but also of reduced apoptosis. The balance between proliferation and apoptosis is crucial in determining the overall growth or regression of the tumor in response to chemotherapy, radiotherapy and, more recently, hormonal treatments. All of these act in part by inducing apoptosis^41^–^43^ and inhibiting cell proliferation. The molecular understanding of cell proliferation and apoptosis signaling (Fig. 3) could allow individually tailored treatments to maximize tumor regression and its efficacy. Cancer cells overcome cell death or apoptosis due to mutation of tumor suppressor genes and impaired apoptotic machinery executing cell death. The apoptotic pathway is controlled by a diverse range of cell signals which may originate either extracellularly (extrinsic inducers) or intracellularly (intrinsic inducers). These signals may positively or negatively induce apoptosis. The extrinsic pathway is activated by binding of ligands such as FasL and TNF to FAS and TNFR, inducing the formation of death induced signaling complex (DISC). DISC in turn recruits caspase-8 through FADD (Fas-Associated Death Domain) and promotes the cascade of procaspase activation and finally caspase-7 leading to apoptosis. The intrinsic pathway is triggered by various extracellular and intracellular stresses, such as growth-factor withdrawal, hypoxia, DNA damage and oncogene induction. Signals that are transduced in response to these stresses converge mainly on the mitochondria. A series of biochemical events is induced that results in the permeabilization of the outer mitochondrial membrane, the release of cytochrome-c and other proapoptotic molecules, the formation of the apoptosome, a large protein complex that contains cytochrome-c, apoptotic protease activating factor 1 (APAF1) and caspase-9 and finally caspase-3/7 activation and cell death.^44^ There is a cross talk between extrinsic and intrinsic pathway. Activated caspase-8 cleaves Bid, and the COOH-terminal part translocates to mitochondria where it triggers cytochrome-c release.^45^,^46^ Apoptosis is mainly governed by the net ratio of pro-apoptotic and anti-apoptotic Bcl-2 family members (Table 1). Higher Bax/Bcl-2 ratio predicts good response for chemotherapy in breast cancers, indicating apoptosis. There are at least 19 Bcl-2 family members that have been identified in mammalian cells, with several others found in viruses.^47^ They possess at least one of the four conserved motifs known as Bcl-2 homology domains (BH1 to BH4).^48^,^49^ Most anti-apoptotic members contain all four of these domains. Some pro-apoptotic Bcl-2 family proteins possess only the BH3 domain, which is essential for their function,^50^,^51^ indicating the role of BH3 domain in apoptosis. These pro- and apoptotic Bcl-2 family members will be studied in detail to understand and unveil the chemoresistance in breast cancers.

### Apoptosis regulators

Prognostic markers of breast cancers (Table 2) include apoptotic regulators, growth factors, proteases and topoisomearse. Apoptotic regulation is controlled by Bcl-2 family of proteins. The expression of Bcl-2 protein has been shown to suppress apoptosis in response anticancer drugs.^52^,^53^ Therefore, Bcl-2 may mediate chemoresistance in some patients and Bcl-2 could be a target for the development of new anticancer therapy. Inhibition of expression of Bcl-2 family members by antisense oligonucleotide or dominant negative inhibitor Bcl-xs has already been shown to promote apoptosis and to sensitize cells to chemotherapy-induced apoptosis.^54^ Despite the antiapoptotic and chemoresistant effects favoring tumor survival, Bcl-2 prolongs cell cycle and decreases tumor cell proliferation, and these functions may account for the association of Bcl-2 in favorable breast cancer outcomes. In humans Bcl2 is expressed in about 80% of breast cancers^55^,^56^ in women with primary tumors, having either node positivity or negativity and is correlated with the expression of ER and PR. Zhang et al^57^ observed that Bcl-2 gradually decreases during the development of breast cancer, i.e. from a normal epithelium (96%) to intraductal carcinoma (79%), and from intraductal to invasive carcinoma (45%), and reverse is observed in case of p53 expression. Consistent with these findings, Haldar et al^58^ reported that transfection of mutant p53 into a p53-wild-type breast cancer cell line suppressed expression of Bcl-2. Major correlations between Bax protein and outcome have not been observed, although in patients with breast cancer, a reduced expression of Bax was correlated with a poor response to chemotherapy and metastatic breast cancer.^59^,^60^ Restoration of Bax expression in breast cancer cell lines increased sensitivity to cytotoxic drug therapy and also suppressed tumorigenesis.^61^,^62^ However, in breast cancers, no correlation between the percentages of Bax and p53-immunopositive tumor cells was observed when examined as either dichotomous or continuous variables, although p53 binds with Bax gene promoter and hence acts as transcriptional activator.^63^ Moreover Bax immunostaining was not significantly correlated with HER-2, cathepsin D or S phase fraction, but was positively associated with Bcl-2. The other antiapoptotic proteins related to Bcl-2 are Bcl-xL and Mcl-1. The remarkable structural similarity between the Bcl-2 and Bcl-x genes suggests that they have evolved from a common ancestral gene by way of gene duplication.^64^ Through alternate splicing, Bcl-x can encode several proteins, including the Bcl-xL (long) protein which inhibits apoptosis and a shorter isoform Bcl-xS which promotes apoptosis by acting as a trans-dominant inhibitor of Bcl-2 and Bcl-xL. Bcl-xL inhibits cytochrome-c redistribution from inter membrane space of mitochondria to cytosol by blocking both membrane hyperpolarization and mitochondrial swelling in response to several sets of apoptotic stimuli.^65^ Moreover, recent studies report that Bcl-xL protein interacts with Apaf-1 to inhibit the caspase activation involved in apoptosis,^66^ increases metastatic potential of breast cancer. Increased levels of Bcl-xL expression were found in a subset of primary human breast carcinomas, mainly in undifferentiated histological grade (HG) III tumors.^67^,^68^ In the breast, their overexpression could protect breast cancer cells from p53-mediated apoptosis.^69^ More recently BAG-1, a multi functional protein that blocks apoptosis, has been correlated with improved survival in early stage breast cancer. Again, this seemingly contradictory finding in an inhibitor protein reflects the complex mechanisms in which these proteins work; BAG-1 is known to interact with other members of the Bcl-2 family as well as heat shock proteins and estrogen receptors. Another member of pro-apoptotic Bcl-2 family consists of Bad that bind to anti-apoptotic Bcl-2 and Bcl-xL, thus induce apoptosis. In fact, in normal human breast cells, Bad levels are relatively high in comparison with those in other organs,^70^ suggesting that Bad might have a special role in the breast. Bad is regulated by growth factors and cytokines, stimulating Bad phosphorylation on specific serine residues: S112, S136, S155 and S170. Phosphorylation of Bad on these residues promotes its binding to, and subsequent cytosolic sequestration by, 14-3-3 proteins. Thus phosphorylation at serine residues prevents Bad from associating with Bcl-2 or Bcl-xL on the mitochondrial outer membrane, leaving these proteins free to exert their anti-apoptotic function.^71^ Indeed, Gilmore et al^72^ showed that transient Bad overexpression enhances the sensitivity of mammary cells to apoptosis, perhaps because the S112 and S136 kinases become limiting. On the other hand, primary cultures of Bad−/− mammary cells are no longer sensitive to ZD1839-induced apoptosis, suggesting that Bad might be the important pro-apoptotic effector downstream of the EGFR. Survivin, a member of the inhibitor of apoptosis (IAP) gene family, containing only a single baculoviral IAP repeat (BIR) domain. Survivin is overexpressed in most human cancers, including breast, but is not expressed in normal tissue. 84% of breast carcinoma showed nuclear survivin expression.^73^ Survivin is associated with more aggressive behavior and decreased survival in a variety of tumor types. It regulates the G2/M phase of the cell cycle by associating with mitotic spindle microtubules, and it directly inhibits caspase-3 and caspase-7 activity. There is a gradual rise in the expression of caspases 3, 6 and 8 and the expression of these caspases are strongly associated with increase in apoptosis, with a direct relationship between the apoptotic index and the histological aggressiveness of a breast lesion.^74^ Expression of genes such as *p53* and *c-erb-B2* are also increased in higher grade tumors. It is possible that such genes may be responsible for the increased apoptotic rate observed in these tumors.

### Molecular targets and chemotherapeutic drugs

Breast cancer is characterized by overexpression of ER/PR, EGFR, HER-2 and VEGFR-2, along with Bcl-2 and downreguation of p53. At present, the circle of agents with an established chemopreventive effect is restricted to tamoxifen and raloxifene in breast cancer. However, in recent years, there has been an exponential increase in the study of agents (Table 3) that have a chemopreventive potential against molecular targets of breast cancer. The treatment for cancer cells that may have spread beyond the breast, lymph nodes and nearby tissues is a combination of either hormone therapy (antiestrogen) and/or chemotherapy. Metastatic breast cancer (MBC) develops in 30%–40% of patients with breast carcinoma and patients with MBC have a median survival of about 2 years and, therefore, new anticancer agents are urgently required.^75^ Chemotherapeutic drugs are applied to patients depending upon size, lymph node status, ER and PR, and HER-2/neu over-expression. Herceptin is a monoclonal antibody targeting HER-2 overexpression breast cancer cells. It induces HER-2 receptor downmodulation and, as a result, inhibits Ras/Raf/MAPK and PI3-K/Akt pathways leading to inhibition of cell proliferation and apoptosis and also blocking cell cycle progression by inducing the formation of p27/Cdk2 complexes.^25^,^76^,^77^ It is found that addition of herceptin along with other chemotherapeutic drugs for early stage HER-2 positive breast cancers reduced the risk of recurrence and death by 52% and 33% respectively, compared to chemotherapy alone.^78^,^79^ There is another humanized monoclonal antibody, pertuzumab (2C4) that binds to different epitope than that of herceptin. It mainly inhibits HER-2 heterodimerization with HER-1, HER-3 and HER-4 and thus inhibits downstream cell proliferation pathways of HER-2 based heterodimers in cells with low or high expression of HER-2 receptors.^25^ Bevacizumab (Avastin) is a monoclonal antibody against VEGF, thus help in inhibiting in angiogenesis. It is approved by FDA in 2008 and used in metastatic breast cancer as adjuvant therapy along with 5- florouracil (5-FU), oxaliplatin. The most common chemotherapeutic drugs recommended to be used in combination in early breast cancer are cyclophosphamide, methotrexate, fluorouracil, doxorubicin (adriamycin), epirubicin, paclitaxel (Taxol), and docetaxel (Taxotere). Paclitaxel (PAC) and docetaxel (DOC) are clinically effective anti neoplastic agents and excellent choices for the first- and second-line treatment of patients with MBC.^80^,^81^ Taxanes bind to β-subunit of tubulin, retard microtubule depolymerization, impair mitosis, and block progression through the cell cycle. In addition, the taxanes inactivate the Bcl-2 protein and induce apoptosis in breast cancer cells in vitro.^82^,^83^

Better understandings of breast cancer at molecular level helps in designing new potential anticancer drugs that target specific receptors and tumors, thus increasing the efficiency and having lesser side effects than conventional chemotherapy. Such targeted therapies as adjuvant therapy benefits the patients in advanced diseases, delay the time of cancer reoccurrence as well as reverses the hormone- and chemo-resistance. Lapatinib, a dual tyrosine kinase inhibitor of EGFR and HER-2, inhibits autophosphorylation of tyrosine kinase residue and exerts greater biologic effects in inhibiting signaling pathways of cell proliferation and inducing apoptosis than inhibition of either receptor alone.^84^ It has been approved by FDA in 2007 for use in patients with advanced metastatic breast cancer along with chemotherapeutic drugs like taxanes, herceptin, and capecitabine. It is effective in delaying cancer progression in HER-2 positive advanced metastatic breast cancer women who have become resistant to Herceptin.^85^ There is another class of drugs that fall under hormone therapy. They can be further divided into selective estrogen receptor modulators (SERMs) and aromatase inhibitors (AIs). Tamoxifen (Nolvadex) and raloxifene compete with estrogen in binding ER. Upon binding the receptor complex may act as agonist by binding with co-activators and enhance the estrogenic signaling responses or antagonist by binding with co-repressors and thus inhibiting the estrogenic responses. Moreover the outcome of the response is also governed by the type of tissue and the RTK by enhancing the estrogenic responses. Thus the final outcome is governed by the balance between antagonist and agonist effect of SERMs.^86^ Tamoxifen is used in ER+ metastatic breast cancer as adjuvant therapy in a pre and post menopausal women. It is cytostatic drug leads to accumulation at G0/G1 phase perhaps by forming the complex p21/CDK2.^87^ Tamoxifene also leads to endometrial cancer after prolong use, whereas raloxifene doesn’t and it is as effective as tamoxifene and used in treating ER+ invasive breast cancer patients with postmenopausal osteoporosis.^88^,^89^ AIs include anastrozole (Armidex), letrozole (Femara) and exemestane (Aromasin). AIs block the conversion of androstenedione into estrogen and are found to be more effective than tamoxifen in treating post menopausal women with ER+ breast cancers.^90^–^92^ Though, lot of cytotoxic drugs is available, but still challenges in treatment remains. Many tumor cells become resistant to commonly used cytotoxic drugs due to the overexpression of ABC transporters. Two proteins, P-gp (MDR-1, ABCB1) and MRP-1 (ABCC1) have been demonstrated to pump a wide selection of the most commonly used anti-cancer drugs and their overexpression correlates broadly with negative treatment response characteristics in many different forms of cancer. It is also found that genetic alteration or gene amplification of metastasis gene metadherin (MTDH) is associated with more than 40% breast cancer cases with poor clinical outcomes.^93^ These findings establish that anticancer drugs targeting ABC transporters and MTDH can be used in increasing the chemotherapeutic efficacy and reducing metastasis risk.

## Conclusions

The prognosis and over all survival of breast cancer has improved over years, with current 10 year relative survival of about 70% in western population.^94^,^95^ Positive lymph node status, tumor size, PR and ER, HER-2 are the most reliable prognostic factors in predicting overall as well as disease free survival of breast cancer patients.^96^ The knowledge of individual prognostic marker remains poorly understood at molecular level in breast cancer prognosis and prediction of particular treatment regimens and its overall survival. Gene profiling, the future diagnostic tool, help in clustering and segregation of genes based on disease outcome and correlation with established biomarkers ER/PR and HER-2. It has the potential to find new biomarkers and solve the molecular basis of chemoresistance. It is expected that more patient samples and gene profiling datas will help in validation of various prognostic factors i.e. EGFR-1, cell cycle molecules, apoptosis related proteins, VEGF, sVEGFR, matrix metalloproteinase (MMP)-2 and MMP-9, tissue inhibitors of metalloproteinases, plasminogen activators and inhibitors, etc. that are currently in investigation for primary and advanced breast cancer. The increasing number of biomarkers associated with breast cancers should yield a better test for prognosis and thus increase the screening sensitivity between healthy and cancer at early stages. Moreover, it’ll also help in designing cancer therapies and drugs that are tailored to unique characteristics of each individual and tumor. Such rational therapies will have less adverse effects than conventional therapy and will have better outcome in overall and disease free survival.

## Figures and Tables

**Table 1.** Bcl-2 family members.

**Anti-apoptotic**	**Pro-apoptotic**
BCL-2	BAX
BCL-XL	BAK
MCL-1	BOK
A-1/BFL-1	BAD
BCL-W	BID
BOO/DIVA	BIK
NR-13	BLK
	HRK
	BIM
	BNIP3
	NIX
	NOXA

Esteva and Hortobagyi.^47^

**Table 2.** Bio-molecular markers of breast cancers.

**Markers**	**Occurrence (%)**	**Treatment**	**References**
EGFR	17%	Chemotherapy	97
HER-2	25%	Monoclonal Humanized antibody	25,76
VEGFR	64% (Invasive breast cancer)	Chemotherapy	34,98
ER and PR	70%–80%	Endocrine therapy	99,10
BRCA1 and BRCA2	2%–4%	Chemotherapy and Radiotherapy	100
P53	25%–30%	Chemotherapy	101–103
P21 (>10% of cells positive)	65%	Chemotherapy	104
Bcl-2	80%	Chemotherapy, antisense RNA	55,56
Topoisomerase II α	>50.2%	Chemotherapy: Anthracycline	105
uPA and PAI-1	46%	Adjuvant chemo or endocrine therapy	106,107
Caspase-3	75.2%	Chemotherapy	108
*TIP30/CC3	83.3%	Genetherapy	109,110
**Ki-67	56%	Chemotherapy and endocrine therapy	111,112

* TIP30/CC3 is metastatic tumor suppressor gene. Decrease in expression of TIP30 is observed in 48% of breast cancer cases and 83.3% of TIP30 negativity tumors had lymph node metastasis and vascular invasion.

** Ki-67 cut off value for primary (P) tumors is 10% and 15% for metastatic (M) tumors. P−/M− = 44%, P+/M+ = 27.3%, 14%, P−/M+ = 21.1%, P+/M− = 7.2%.

**Abbreviations:** uPA, urokinase plasminogen activator; PAI-1, plasminogen activator inhibitor-1.

**Table 3.** Drugs used for breast cancer treatment.

**Drugs**	**Molecular targets**
Tamoxifen	ER
Raloxifene	ER
Steroidal AI: Exemstane	Aromatase/Estrogen
Non-steroidal AI: Anastrozole and Letrozole	Aromatase/Estrogen
Herceptin	HER-2
Pertuzumab	HER-2
Bevacizumab	VEGF/VEGFR
Gefitinib	EGFR
Zactima	VEGFR/EGFR
Doxorubicin	DNA, Topoisomerase II complex
Taxane (Paclitaxel, docetaxel)	β subunit of tubulin, Bcl-2
5-Fluorouracil, capecitabine (prodrug)	Thymidylate synthase
